# Mast cells in colorectal cancer tumour progression, angiogenesis, and lymphangiogenesis

**DOI:** 10.3389/fimmu.2023.1209056

**Published:** 2023-07-11

**Authors:** Xiaoxin Liu, Xinyu Li, Haotian Wei, Yanyan Liu, Ningxu Li

**Affiliations:** ^1^Department of Nephrology, Liyuan Hospital, Tongji Medical College, Huazhong University of Science and Technology, Wuhan, Hubei, China; ^2^Department of Nephrology, Tongji Hospital, Tongji Medical College, Huazhong University of Science and Technology, Wuhan, Hubei, China

**Keywords:** colorectal cancer, mast cells, angiogenesis, lymphangiogenesis, immune cells, cancer

## Abstract

The characteristics of the tumour cells, as well as how tumour cells interact with their surroundings, affect the prognosis of cancer patients. The resident cells in the tumour microenvironment are mast cells (MCs), which are known for their functions in allergic responses, but their functions in the cancer milieu have been hotly contested. Several studies have revealed a link between MCs and the development of tumours. Mast cell proliferation in colorectal cancer (CRC) is correlated with angiogenesis, the number of lymph nodes to which the malignancy has spread, and patient prognosis. By releasing angiogenic factors (VEGF-A, CXCL 8, MMP-9, etc.) and lymphangiogenic factors (VEGF-C, VEGF-D, etc.) stored in granules, mast cells play a significant role in the development of CRC. On the other hand, MCs can actively encourage tumour development via pathways including the c-kit/SCF-dependent signaling cascade and histamine production. The impact of MC-derived mediators on tumour growth, the prognostic importance of MCs in patients with various stages of colorectal cancer, and crosstalk between MCs and CRC cells in the tumour microenvironment are discussed in this article. We acknowledge the need for a deeper comprehension of the function of MCs in CRC and the possibility that targeting MCs might be a useful therapeutic approach in the future.

## 1 Introduction

According to estimates, there were more than 1.9 million new cases of CRC and 935,000 deaths from this disease worldwide in 2020 (1). A thick connective tissue interstitial milieu composed of epithelial cancer cells, extracellular matrix (ECM), fibroblasts, endothelial cells, and immune cells is one of the key characteristics of CRC (2). More people have become aware of the close association between colorectal cancer development and the immune system in recent years. Tumourigenesis, which is characterized by genomic instability, dysregulated gene expression, and anomalies in the epigenome, requires the coordinated action of several processes (3). However, altered cells are regularly removed through immune surveillance, which stops tumour development (4). Through intricate methods, many immunosuppressive cells can evade immune system detection, allowing for ongoing tumour growth. Tumour development requires angiogenesis, and lymphangiogenesis is crucial to lymph node metastasis (5, 6). Mast cells are found in all vertebrates (7) and were described and originally named by Paul Ehrlich in 1878 (8). Westphal first postulated that MCs played a protumourigenic role in 1891 (9). MCs are derived from hematopoietic stem cells that are CD34+ (10) and CD117+ (KIT) and can be separated into two groups based on their composition: M(T) and M (Tc). Mc(T) granules are rich in trypsin and are mainly located in the mucosa of the gastrointestinal and respiratory tracts (11, 12). On the other hand, Mc(TC) particles contain trypsin, chymotrypsin, and carboxypeptidase and are mainly located in the submucosa, connective tissue, near blood vessels, and lymphatic vessels (12). Mast cells, which act as the barrier between the host and the outside environment, can enhance host defense against infections by controlling the immune response (13). Mast cells are also critical immune cells that can secrete cytokines that alter tumour growth in the inflammatory milieu, and they play a significant role in hypersensitivity, particularly type I hypersensitivity. Proteases (including trypsin and chymase), histamine, cytokines, chemokines, and angiogenic factors are among the bioactive mediators found in the cytoplasm of mast cells (11, 14). The conventional pathway, which is mediated by IgE binding to the FcRI receptor on the surface of mast cells, is the most well-known method of initiating MC degranulation; however, the activation of C3a and C5a in the inflammatory milieu can directly induce MC degranulation (15). Mast cells secrete a variety of bioactive mediators that can inhibit and promote tumours (16). The heterogeneity of mediators released by MCs depends on the tissue, environment, and different pathways that activate MCs, such as IgE-dependent activation, IgG immune complex crosslinking with FcγRIII, C3a and C5a complement receptor activation, stem cell factor (SCF)-bound c-kit receptor, and TLR 2 (toll-like receptor 2) activation (17). It is interesting to note that mast cells produce naturally occurring and immune-mediated proangiogenic factors that help blood vessels form (e.g., VEGF-A, endothelin-1, GM-CSF, and CXCL 8) (18, 19). In the tumour microenvironment, these cells can also release compounds that promote lymphangiogenesis, such as VEGF-C and VEGF-D (9). The role of MCs in CRC is still controversial and uncertain. However, understanding the molecular mechanisms underlying the interaction between MCs, cancer cells, and other elements of the tumour microenvironment may help in the search for a way to interfere with the interaction between cancer cells and other cells to stop the growth and reproduction of cancer cells.

## 2 MC growth and biological function

Half a billion years ago, the innate immune system included MCs, which exerted antiparasitic and antibacterial effects in the host, and they can be found in the hemolymph of the ascidian (sea squirts) (20, 21). MCs are mainly derived from the myeloid lineage of bone marrow hematopoietic stem cells (22). CD34^+^/CD117 ^+^pluripotent MC progenitors (MCps) leave the bone marrow, migrate and colonize target tissues (e.g., gastrointestinal tract, skin, perivascular space, perineural connective tissue and respiratory tract) via specific integrin and chemokine receptors (23–26). Subsequently, in response to stem cell factor (SCF), IL-3, IL-4, IL-9, IL-10, TGF-β, and IL-33, MCps develop into MCs with dense granules (27–32). A recent study showed that MCps and mature MCs express some of the same chemokine receptors, such as CXCR4 and CCR1, suggesting that mature mast cells can still migrate to other tissues after maturation (33). MC surface receptors bind to tumour-derived cytokines and growth factors, which recruits these cells into the tumour microenvironment. For example, SCF produced by tumour cells bind to the c-Kit receptor on mast cells (34–36). Several chemokines derived from tumours (CCL2, CCL5, CCL11, CCL15, CXCL1, CXCL2, CXCL10 and CXCL12) can activate mast cell receptors (CCR2, CCR3, CXCR2, CXCR3 and CXCR4) to induce MC migration (34, 37–43). On the other hand, VEGF, platelet-derived growth factor AB (PDGF-AB), basic fibroblast growth factor (bFGF), and adrenomedullin (AM) produced by tumour cells can induce mast cell chemotaxis (44). Mast cells play an important role in innate and adaptive immunity (45). Mast cells are among the first cells to come into contact with pathogens, and so they are reliable prerequisite cells for preventing infection in humans (46). Mast cells can fight pathogens through direct antibacterial, antiviral and antiparasitic effects (e.g., the release of multiple antimicrobial peptides, killing bacteria after binding to complement or IgG Fc receptors, and endocytosis) (47–50). The more important role of mast cells in innate immunity is to recruit other innate immune cells, such as neutrophils, eosinophils and macrophages, to the site of infection (51–53); thus, multiple immune cells come together to better clear pathogens. On the other hand, several costimulatory molecules (CD40L, OX40L, CD80, and CD86) on mast cells and the various cytokines (IL-4, IL-5, IL-6, IL-13 and IL-33) they produce can influence the biological behavior of TH2 cells and B cells and modulate regulatory T cells (Tregs), thus regulating adaptive immunity (46). For example, MC-derived IL-25, IL-33, and TSLP can activate antigen-presenting cells (e.g., DCs) to eventually regulate the functional status of TH2 cells (54). In conclusion, mast cells play an important role in the protection of human health and in the pathophysiology of various diseases (e.g., cancer), IgE-driven allergic diseases, cardiovascular diseases, autoimmune diseases and cancer) (55).

## 3 The controversial role of MCs in cancer

Depending on the kind, stage, grade, and size of the tumour, as well as their microanatomical placement inside the tumour, tumour-associated mast cells (TAMCs) can have pro- or antitumourigenic effects on the host (56). However, in a some circumstances, these cells do not seem to have any impact on the development or progression of tumours (57–59). The protumour activity of MCs and the link between TAMCs and poor clinical outcomes in a variety of cancers, including Hodgkin lymphoma, gastric cancer (GC), pancreatic cancer, cholangiocarcinoma, and bladder cancer, are supported by a number of research investigations. MC infiltration is associated with a worse prognosis and lower relapse-free survival rates in Hodgkin’s lymphoma (60–62). *In vitro* experiments showed that Hodgkin’s lymphoma could promote tumour cell proliferation through CD30L-CD30 interactions between mast cells and cancer cells (60, 63). Similar results have been observed in gastric cancer, in which the presence of tumour-infiltrating MCs is related to tumour progression and independently predicts a lower overall survival rate (64–66). Tumour-derived adrenomedullin (ADM) stimulated mast cell production of IL-17A, which can boost GC cell proliferation and block GC cell death *in vitro* (66). Intriguingly, pancreatic cancer cells have been shown to attract MCs to the tumour microenvironment. MCs then aid in tumour cell proliferation and invasion, hastening disease development (67). MC infiltration is enhanced along with carcinogenesis in cholangiocarcinoma (68) and bladder cancer (69). Mast cells in these tumours have protumourigenic effects by influencing tumour biology, including angiogenesis, lymphangiogenesis, invasiveness, and tumour cell proliferation, which ultimately results in a poor prognosis for patients.

Research linking the presence of mast cells to various tumour types seem to be contradictory. High concentrations of peritumoural mast cells were linked to a poor prognosis in prostate cancer, although mast cell densities inside tumours were an independent favorable prognostic predictor (70–72). The different anatomical placements of the mast cells might be the cause of these opposing effects (peritumoural vs. intratumoural). The accumulation of mast cells in the peritumoural compartment during the development of a castration-resistant prostate tumour ultimately resulted in tumour palindromia (70). Mast cells have been linked with a favorable prognosis in breast carcinomas in some studies (73–77) but not all of them (78–80). There is also a high degree of intertumour and intratumour heterogeneity among patients (81). In lung adenocarcinoma, a higher MC count was associated with poor prognosis in stage I NSCLC (82). In contrast to another study, a low density of peritumoural mast cells was associated with a worse prognosis in stage I lung adenocarcinoma (83). In skin cancers, human and animal studies targeting the function of mast cells and their mediators have obtained controversial outcomes (84). Mast cell-derived serine proteases inhibit the growth of melanoma (85); however, data have also reported that MCs are associated with poor prognosis (86) and resistance to immune therapy (87).

## 4 TAMCs in tumour angiogenesis and lymphangiogenesis

Angiogenesis, which is the growth of new blood vessels, is essential to many physiological processes that take place as the human body develops (5). Lymphangiogenesis, which is the development of new lymphatic vessels, is a process that is active in some diseases (wound healing, chronic inflammation, tumour metastasis, etc.) (88). The ratio of substances that stimulate angiogenesis and lymphangiogenesis to those that prevent it determines the rate (89). It is interesting to note that the regulation of lymphangiogenesis and angiogenesis is mediated by innate and adaptive immune cells, including mast cells (90). In 1971, Judah Folkman proposed that angiogenesis was necessary for the growth of tumours (91), and he later proposed that mast cells may be a major source of substances that promote angiogenesis (92). Similarly, tumour-associated lymphangiogenesis plays an integral role in lymph node metastasis and tumour progression (93). Many experiments have demonstrated that pro-angiogenic factors (VEGF-A, VEGF-B) (94–97), and pro-lymphangiogenic factors (VEGF-C and -D) (43, 94) are synthesized by mast cells. VEGF receptor 2 (VEGFR-2) is expressed by blood endothelial cells (BECs), and VEGF-A activates it to carry out its intended tasks (98). Since VEGFs induces mast cell chemotaxis by binding to the VEGFR-1 and VEGFR-2 receptors on their surface, mast cells serve as both the source and the target (89, 99). Lymphangiogenesis depends on VEGF-C and VEGF-D binding to their receptor VEGFR-3 (100, 101). In addition to playing a crucial switching role in tumour-associated angiogenesis (102), angiopoietins (Angs) and their endothelial cell receptor Tie2 can encourage the growth of lymphatic vessels (103). Ang1 expression by pericytes is essential for vascular maturation, Ang2 is produced by ECs, and both of these factors agonize Tie2 under certain conditions (104, 105). Mast cell-derived chymotrypsin converts Ang1 to Ang2 and accelerates angiogenesis (106). Tie1 and Tie2 are expressed on the surface of human lung mast cells (HLMCs), and the binding of Ang1 to Tie2 can cause mast cell migration (107). When stem cell factor (SCF) binds to c-KitR on the MC surface, the c-KitR pathway is activated, inducing MC degranulation and the release of trypsin and pro-angiogenic cytokines (such as VEGF, PDGF, and FGF-2) (108, 109). Trypsin produced by mast cells can directly stimulate endothelial cell growth (110) or indirectly activate matrix-metalloproteases (MMPs) and plasminogen activator (PA) to degrade the extracellular matrix, providing space for neovascularization and facilitating the invasion and metastasis of cancer cells (36, 111). Moreover, trypsin can activate protease-activated receptor 2 (PAR-2) (112), which is expressed on endothelial cells in the blood vessel wall (113). When PAR-2 is activated, endothelial cells multiply, and proangiogenic chemicals, including IL-6 and granulocyte macrophage colony-stimulating factor, are released (114). Notably, IL-1 can trigger human mast cells to produce CXCL8/IL-8, effectively increasing angiogenesis (115). Extracellular adenosine is elevated in the tumour microenvironment because of hypoxia, and this factor can activate adenosine receptors on the surface of mast cells, which increases the production of VEGF and CXCL8/IL-8 (116), ultimately promoting angiogenesis and lymphangiogenesis.

## 5 Mast cells and prognosis in CRC

The role of mast cells in colorectal cancer progression is controversial. Many researchers have investigated the correlation between MCs and CRC patient prognosis (**Table 1**). Some authors believe that MCs are correlated with a good prognosis (22, 117, 118), while others believe that mast cells are not associated with prognosis (59, 119, 120), but most studies suggest that mast cells are associated with reduced survival rates (2, 121–123).

**Table 1:** Role of MCs in the outcome of colorectal cancer.

Publication	Disease stage	Methods of MCs identification	Localization	Prognosis
Mehdawi et al. (117)	All TNM stages	Tryptase and Chymase	Intratumoural/ peritumoural	Positive
Elezoglu et al. (118)	All TNM stages	Toluidine blue	Intratumoural/ peritumoural	Positive
Song et al. (22)	All TNM stages	Tryptase	Intratumoural/peritumoural	Positive
Xia et al. (59)	IIIB stage	Tryptase	Intratumoural/peritumoural	No relationship
Xia et al. (119)	All TNM stages	Tryptase and Chymase	Intratumoural/peritumoural	No relationship
Zhao et al. (120)	All TNM stages	Flow cytometric analysis	Intratumoural/peritumoural	No relationship
Zhao et al. (120)	All TNM stages	Flow cytometric analysis	Blood samples	Positive
Wu et al. (2)	All TNM stages	Tryptase	Intratumoural/peritumoural	Negative
Shinsuke et al. (121)	IV stage	Tryptase	Peritumoural	Negative
Malfettone et al. (122)	All TNM stages	Tryptase	Peritumoural	Negative
Mao et al. (123)	All TNM stages	CIBERSORT/Tryptase	Intratumoural/peritumoural	Negative
Gulubova et al. (124)	All TNM stages	Tryptase/Toluidine blue	Intratumoural/peritumoural	Negative

Song et al. analyzed pathological tissues from 164 CRC patients and found that high mast cell density (MCD) levels were significantly associated with longer overall survival of patients (22). Mehdawi et al. (117) observed that fewer MCs were found in cancer tissue from 72 CRC patients than in normal colon tissue and that patients with relatively higher MCD in cancer tissue had a significantly longer overall survival.

However, Mao et al. (123) confirmed that MCD was an independent prognostic factor, and low tumour infiltration MCD was associated with increased overall survival, which may be due to the association of low MCD with a stronger immune response to aid prolonged survival in patients with a low MCD, MCD has also been shown to predict survival in stage II and III CRC patients treated with adjuvant chemotherapy. Suzuki et al. (121) reported that high peritumoural MC infiltration was a significantly unfavorable prognostic factor in 135 patients with colorectal liver metastasis (CRLM) who underwent liver resection, and the number of MCs in liver metastatic lesions could significantly predict the prognosis of CRLM patients and was an indication for treatment. Wu et al. (2) showed that MC infiltration was significantly associated with sex, lymph node status, and American Joint Committee on Cancer stage, and high MC infiltration can serve as an independent biological marker to predict poor survival in colorectal cancer patients. Thus, the identification of patients with high risk of tumour progression can be achieved by immunohistochemical analysis of tumour-infiltrating mast cells, thus optimizing personalized treatment for CRC patients.

In contrast, Xia et al. (119) observed that mast cell counts in adjacent normal colon mucosa were associated with pathological classification, distant metastasis, and liver metastasis but were not a prognostic factor. Instead, mast cell counts in the invasive margin showed no correlation with clinicopathological parameters or overall survival. Zhao et al. (120) reported that circulating mast progenitor cell (MCp) levels are low in CRC patients and are significantly associated with CRC progression, and the frequency of MCps may be an independent indicator of the aggressiveness of CRC in patients and may be used to distinguish between patients with early and advanced CRC. However, mast cells in tumour tissue are not associated with CRC progression.

These conflicting results stem in part from the high heterogeneity of studies on MCs and CRC. For example, different tumour regions have been examined in many studies on MCs and CRC, and some studies examined the surrounding tumour regions, while others examined the central tumour areas, and many studies did not report the tumour regions examined, making it difficult to compare. However, there is a link between the distribution of MCs and the prognosis of CRC patients. MC infiltration was defined as a favorable independent prognostic factor in CRC patients (124); however, a large number of MCs confined to the tumour periphery is associated with tumour progression (121, 125, 126). On the other hand, stage IIIB colon cancer was shown by Xia et al. (59) to have mast cell numbers that varied depending on where in the tumour they were located, and the interstitium of primary colon cancer had fewer mast cells than the neighboring mucosa.

In addition to the localization of mast cells, MCs from different tissues were analyzed by transcriptional profiling, and MCs showed large transcriptional heterogeneity between different tissues (127). MC degranulation status also plays an important role in the prognosis of CRC patients. A recent study showed that the proportion of degranulated mast cells (observed by morphology) was increased in patients with metastatic colorectal cancer, while the proportion of intact mast cells was increased in the nonmetastatic group (128). This may be related to the tumourigenic activity of some products released during MC degranulation.

Recently, it was found that in NSCLC, TAMCs were divided into 2 subgroups based on alphaE integrin (CD103) expression, and CD103+ cells were more likely to interact with T cells and were closer to cancer cells, thus emphasizing the nonnegligible heterogeneity of MCs in cancer (129). In most studies, however, characterization of the MC phenotype was not described in detail.

Therefore, to better understand how MCs affect the prognosis of CRC patients, it is important to focus not only on MC counts but also on understanding their localization, detection methods, degranulation status, degranulation products, and phenotype. For example, we could apply new multiomics, single-cell sequencing and imaging mass cytometry technologies to examine colon cancer-associated MCs and provide a better understanding of the various biological behaviors of mast cells in the tumour microenvironment.

## 6 Cross-talk between MCs, other immune populations, and colon tumour cells

**Figure 1:**
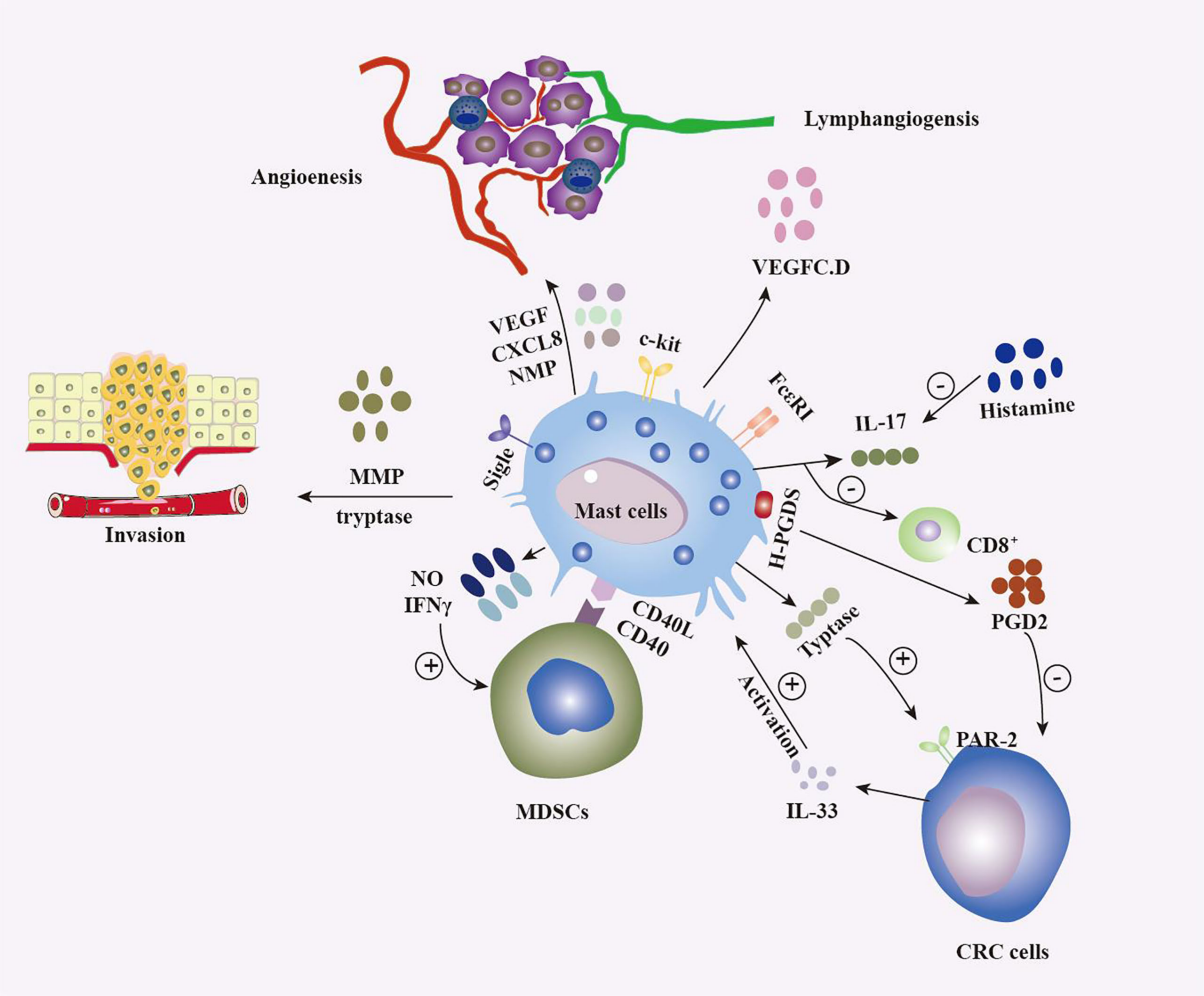
Multiple roles of MCs in colorectal tumours. The release of multiple factors, such as vascular endothelial growth factor, IFN, CXCL8, histamine, nitric oxide, and PGD2, as well as interactions with various populations of the immune system, including CD8+ T cells (130) and MDSCs (131), result in MCs shaping the tumour microenvironment (TME) in different ways and exerting antitumour and protumour effects depending on the context. MCs influence tumour aggressiveness through the release of trypsin and MMPs (111), among other substances. On the other hand, colon tumour cells influence the biological behavior of MCs by various means, such as the release of IL-33 (132) and the activation of Sigle (133), which is surface receptor of MCs.

In recent years, an increasingly close link between the development of colorectal cancer and the immune system has been recognized (**Figure 1**). Immune cells in the tumour microenvironment can influence tumourigenesis and progression and are associated with patient survival (134–136). Zhang et al. (137) confirmed an indicator of immune cell infiltration that included five types of immune cells (resting memory CD4 T cells, M0-M2 macrophages and activated mast cells), and the characteristics of these cells can predict overall survival in late-stage CRC patients. Among these 5 types of immune cells, resting memory CD4 T cells and M0-M1 macrophages are protective factors, and M2 macrophages and activated mast cells are detrimental factors.

Ducroc et al. (113) demonstrated that PAR-2 was expressed in several colon cancer cell lines, and MC-derived trypsin activation of PAR-2 was significantly associated with cell proliferation. The mitogen-activated protein kinase/extracellular signal-associated kinase (MEKK) and mitogen-activated protein kinase (MAPK) pathways are briefly phosphorylated as a result of PAR-2 activation, which promotes the growth of colon cancer cells by increasing the production of cyclooxygenase-2 (COX-2) and prostaglandin E2 (PGE2) (138). Therefore, the proliferation of CRC cells and the growth of associated blood vessels can be inhibited by trypsin inhibitors (gabexate) and c-KitR inhibitors (imatinib, macitinib) (139, 140). On the other hand, activated c-Kit activates the downstream Wnt/β-catenin signaling pathway (141), and Wnt is abundantly expressed in colorectal tumour cells (142), ultimately activating the β-catenin signaling pathway in mast cells in colorectal tumours, while β-catenin stimulates protease maturation and expression in mast cells, and activated β-catenin mediates bone marrow-derived mast cell support of colon cancer (143). Therefore, by blocking the c-Kit receptor with drugs, the β-catenin signaling pathway in MCs will also be inhibited (144), thus inhibiting tumour growth.

In addition, mast cells promote the development of colorectal cancer through several mechanisms. Activation of mucosal mast cells (MMCs) leads to the recruitment of large numbers of CD11b+Gr1+ inflammatory cells into colonic tissue, and MMCs can regulate the activity of CD11b+Gr1+ cells to promote the development of CRC (145). Mast cells can increase the suppressive properties of splenic-derived monocyte MDSCs through IFNγ and nitric oxide production, and the two cell populations interact with each other through CD40:CD40L cross-signaling, which is an axis that is tasked with forming a proinflammatory microenvironment that leads to the production of mediators (TNFα, IL6, CCL-2) (131). Notably, CCL-2 can mediate the migration and activation of MDSCs in tumours (146). Furthermore, mast cells can induce the migration of MDSCs, which can cause immune escape in tumour cells and further cause tumour development (131). On the other hand, mast cells can upregulate RhoA expression in colon cancer cells to activate the Rho/ROCK signaling pathway in tumour cells (147), leading to increased cell mobility (148) and ultimately promoting CRC invasion. The MAPK pathway mediates cell proliferation and differentiation, and many inflammatory factors can activate protein kinases in the MAPK signaling pathway, such as ERK and JNK, which promote tumour progression (149, 150). Mast cells promote tumour-associated angiogenesis through the MAPK/Rho-GTPase/STAT pathway, leading to the development of colon cancer (147). In the hypoxic microenvironment of colorectal cancer, mast cells synthesize hypoxia-inducible factor-1α (HIF-1α) to ensure their own degranulation potential; thus, MC-derived HIF-1α is associated with the release of inflammatory factors (VEGF, IL-6, TGF-β, etc.), and MCs can promote angiogenesis and tumour metastasis by synthesizing HIF-1α (151).

Mast cells affect the development of colon cancer cells, and tumour cells affect the biological behavior of mast cells. YU et al. (152) showed that transcriptome profiling of combined cultures of HT 29 colon cancer cells and MCs showed active expression of MMP-2, VEGF-A, PDGF-A, COX 2, NOTCH1, and ISG 15 by comparing MCs with controls, which revealed how HT 29 makes MCs tumourigenic in the initial stage. These findings provide a new method to study the difference between MCs associated with colon cancer and MCs in normal tissue with a 3D coculture model (152). Many organs express IL-33, which is a cytokine belonging to the IL-1 family (153). The main producers of IL-33 are nonhematopoietic cells such as endothelial cells, smooth muscle cells, adipocytes, myofibroblasts, and epithelial cells (154, 155). Of note, IL-33 is expressed in the tumour epithelium of human colorectal cancer adenomas and carcinomas, and IL-33 activates mast cells and subepithelial myofibroblasts (SEMFs) to express and release ECM components and remodeling proteins, growth factors and angiogenesis modulators, and cytokines to develop a tissue microenvironment that is conducive to polyposis (132). Siglecs are a class of receptors that resemble immunoglobulins and bind sialic acid, and they come in many isoforms and are mostly expressed on immune cells (156). Siglec-6 is the isoform that is the most highly expressed in human MCs, which also express Siglec-3, Siglec-5, Siglec-6, Siglec-7, and Siglec-8 (157, 158). Yu et al. (133) discovered that Siglec-6 was a functional inhibitory receptor for MCs, and Siglec-6 was upregulated on MCs when colon cancer cells (HT29 and co2) were cocultured with MCs, suggesting that MC activity may be regulated through Siglec-6 in the tumour microenvironment of colorectal cancer and demonstrated Siglec-6 expression on human CRC tissue for the first time.

IL-17 is an inflammatory cytokine that is notably increased in gastrointestinal inflammation and cancer (159). The intestine contains many cells that express IL-17, such as innate-like T cells, αβ and γδ T cells, NKT and NK cells, macrophages, granulocytes and mast cells (160–162). Chen et al. (163) found that in histamine-deficient intestinal immunity, intestinal MCs expressing IL-17 were expanded in response to food allergy, while MCs expressing IL-17 were actively mobilized, recruited MDSCs to the intestinal mucosa and suppressed CD8 T-cell activity. Notably, these susceptibility factors that increase tumourigenesis can be reversed by histamine therapy, and histamine appears to prevent MC polarization into IL-17-secreting cells (163). Food allergy can affect colorectal carcinogenesis through mast cells and needs further study.

Interestingly, there have recently been experiments (130) demonstrating that MCs can promote or hinder CRC development, and this difference may vary depending on the type of stimulus that promotes CRC. Activated MCs reduce the number of CD8^+^ T cells in tumours and promote the progression of colitis-dependent (colitis-associated (CA)-CRC), but they inhibit colitis-independent (sporadic (s)CRC) development (130). On the other hand, Iwanaga et al. (164) demonstrated that mast cells strongly expressed H-PGDS in the inflamed colon, and the release of PGD2 inhibits colitis and CRC generation by attenuating TNF α signaling. Cystatin C is an endogenous lysosomal cysteine protease inhibitor, and serum cystatin C can be used as a marker for the diagnosis of renal dysfunction (165). Serum cystatin C levels are associated with a variety of diseases, including tumours (166). Recently, it was shown that mast cell-derived cystatin C can specifically induce endoplasmic reticulum stress (ERS) in CRC cells, thereby inhibiting CRC development (22).

## 7 Conclusions

Previously neglected MCs are gradually becoming protagonists in tumourigenesis, and increasing evidence demonstrates their importance in tumour prognosis and therapeutic efficacy. Despite this awareness, the role and pathogenic mechanisms of MCs in tumours are still far from understood. This is mainly reflected in the contradictory results of many studies. This is a result of mast cells having context-dependent phenotypes and plasticity, which are sensitive to the suddenly changing microenvironment. In addition, many studies on the association between MCs and CRC merely showed the number or density of mast cells without addressing other important features, such as the degranulation status of MCs, tumour localization, the characteristics of secreted cytokines and proteases, and crosstalk between associated immune cells and colon cancer cells. MC is closely linked to angiogenesis, lymphangiogenesis, and the progression of CRC, and it is likely to provide targets for new therapies in the future. Therefore, we urgently need higher quality studies to fully understand the biological behavior of MCs in CRC patient tumours.

## Author contributions

YL and NL designed the study. XXL wrote the manuscript. HW and XYL consulted relevant materials and drew pictures. All authors contributed to manuscript revision, read, and approved the submitted version.

## References

[1] Sung H Ferlay J Siegel RL Laversanne M Soerjomataram I Jemal A. Global cancer statistics 2020: GLOBOCAN estimates of incidence and mortality worldwide for 36 cancers in 185 countries. CA Cancer J Clin (2021) 71(3):209–49. doi: 10.3322/caac.21660 33538338

[2] Wu X Zou Y He X Yuan R Chen Y Lan N. Tumour-infiltrating mast cells in colorectal cancer as a poor prognostic factor. Int J Surg Pathol (2013) 21(2):111–20. doi: 10.1177/1066896912448836 22649166

[3] Dawson MA Kouzarides T Huntly BJ. Targeting epigenetic readers in cancer. N Engl J Med (2012) 367(7):647–57. doi: 10.1056/NEJMra1112635 22894577

[4] Zitvogel L Apetoh L Ghiringhelli F Andre F Tesniere A Kroemer G. The anticancer immune response: indispensable for therapeutic success? J Clin Invest (2008) 118(6):1991–2001. doi: 10.1172/JCI35180 18523649 PMC2396905

[5] Marone G Granata F. Angiogenesis, lymphangiogenesis and clinical implications. preface. Chem Immunol Allergy (2014) 99:Xi–xii. doi: 10.1159/isbn.978-3-318-02481-4 24217613

[6] Varricchi G Pecoraro A Marone G Criscuolo G Spadaro G Genovese A. Thymic stromal lymphopoietin isoforms, inflammatory disorders, and cancer. Front Immunol (2018) 9:1595. doi: 10.3389/fimmu.2018.01595 30057581 PMC6053489

[7] Mulero I Sepulcre MP Meseguer J García-Ayala A Mulero V. Histamine is stored in mast cells of most evolutionarily advanced fish and regulates the fish inflammatory response. Proc Natl Acad Sci U.S.A. (2007) 104(49):19434–9. doi: 10.1073/pnas.0704535104 PMC2148307 18042725

[8] Varricchi G Galdiero MR Loffredo S Marone G Iannone R Marone G. Are mast cells MASTers in cancer? Front Immunol (2017) 8:424. doi: 10.3389/fimmu.2017.00424 28446910 PMC5388770

[9] Marone G Varricchi G Loffredo S Granata F. Mast cells and basophils in inflammatory and tumour angiogenesis and lymphangiogenesis. Eur J Pharmacol (2016) 778:146–51. doi: 10.1016/j.ejphar.2015.03.088 25941082

[10] Kirshenbaum AS Goff JP Semere T Foster B Scott LM Metcalfe DD. Demonstration that human mast cells arise from a progenitor cell population that is CD34(+), c-kit(+), and expresses aminopeptidase n (CD13). Blood (1999) 94(7):2333–42. doi: 10.1182/blood.V94.7.2333.419k30_2333_2342 10498605

[11] Galli SJ Tsai M. Mast cells in allergy and infection: versatile effector and regulatory cells in innate and adaptive immunity. Eur J Immunol (2010) 40(7):1843–51. doi: 10.1002/eji.201040559 PMC3581154 20583030

[12] de Souza Junior DA Santana AC da Silva EZ Oliver C Jamur MC. The role of mast cell specific chymases and tryptases in tumour angiogenesis. BioMed Res Int (2015) 2015:142359. doi: 10.1155/2015/142359 26146612 PMC4471246

[13] Marshall JS Portales-Cervantes L Leong E. Mast cell responses to viruses and pathogen products. Int J Mol Sci (2019) 20(17):4241. doi: 10.3390/ijms20174241 31480219 PMC6747121

[14] Wedemeyer J Tsai M Galli SJ. Roles of mast cells and basophils in innate and acquired immunity. Curr Opin Immunol (2000) 12(6):624–31. doi: 10.1016/S0952-7915(00)00154-0 11102764

[15] Ali H. Regulation of human mast cell and basophil function by anaphylatoxins C3a and C5a. Immunol Lett (2010) 128(1):36–45. doi: 10.1016/j.imlet.2009.10.007 19895849 PMC2815128

[16] Theoharides TC Conti P. Mast cells: the Jekyll and Hyde of tumour growth. Trends Immunol (2004) 25(5):235–41. doi: 10.1016/j.it.2004.02.013 15099563

[17] Valent P Akin C Hartmann K Nilsson G Reiter A Hermine O. Mast cells as a unique hematopoietic lineage and cell system: from Paul ehrlich's visions to precision medicine concepts. Theranostics (2020) 10(23):10743–68. doi: 10.7150/thno.46719 PMC7482799 32929378

[18] Jeong HJ Oh HA Nam SY Han NR Kim YS Kim JH. The critical role of mast cell-derived hypoxia-inducible factor-1alpha in human and mice melanoma growth. Int J Cancer (2013) 132(11):2492–501. doi: 10.1002/ijc.27937 23161568

[19] McHale C Mohammed Z Gomez G. Human skin-derived mast cells spontaneously secrete several angiogenesis-related factors. Front Immunol (2019) 10:1445. doi: 10.3389/fimmu.2019.01445 31293594 PMC6603178

[20] Voehringer D. Protective and pathological roles of mast cells and basophils. Nat Rev Immunol (2013) 13(5):362–75. doi: 10.1038/nri3427 23558889

[21] Wong GW Zhuo L Kimata K Lam BK Satoh N Stevens RL. Ancient origin of mast cells. Biochem Biophys Res Commun (2014) 451(2):314–8. doi: 10.1016/j.bbrc.2014.07.124 PMC4145527 25094046

[22] Song F Zhang Y Chen Q Bi D Yang M Lu L. Mast cells inhibit colorectal cancer development by inducing ER stress through secreting cystatin c. Oncogene (2023) 42(3):209–23. doi: 10.1038/s41388-022-02543-z 36402931

[23] Komi DEA Redegeld FA. Role of mast cells in shaping the tumour microenvironment. Clin Rev Allergy Immunol (2020) 58(3):313–25. doi: 10.1007/s12016-019-08753-w PMC7244463 31256327

[24] Dahlin JS Hallgren J. Mast cell progenitors: origin, development and migration to tissues. Mol Immunol (2015) 63(1):9–17. doi: 10.1016/j.molimm.2014.01.018 24598075

[25] Ekoff M Nilsson G. Mast cell apoptosis and survival. Adv Exp Med Biol (2011) 716:47–60. doi: 10.1007/978-1-4419-9533-9_4 21713651

[26] Gentek R Ghigo C Hoeffel G Bulle MJ Msallam R Gautier G. Hemogenic endothelial fate mapping reveals dual developmental origin of mast cells. Immunity (2018) 48(6):1160–71.e5. doi: 10.1016/j.immuni.2018.04.025 29858009

[27] Leist M Sünder CA Drube S Zimmermann C Geldmacher A Metz M. Membrane-bound stem cell factor is the major but not only driver of fibroblast-induced murine skin mast cell differentiation. Exp Dermatol (2017) 26(3):255–62. doi: 10.1111/exd.13206 27619074

[28] Elieh Ali Komi D Bjermer L. Mast cell-mediated orchestration of the immune responses in human allergic asthma: current insights. Clin Rev Allergy Immunol (2019) 56(2):234–47. doi: 10.1007/s12016-018-8720-1 30506113

[29] Saluja R Hawro T Eberle J Church MK Maurer M. Interleukin-33 promotes the proliferation of mouse mast cells through ST2/MyD88 and p38 MAPK-dependent and kit-independent pathways. J Biol Regul Homeost Agents (2014) 28(4):575–85. 25620169

[30] Westerberg CM Ullerås E Nilsson G. Differentiation of mast cell subpopulations from mouse embryonic stem cells. J Immunol Methods (2012) 382(1-2):160–6. doi: 10.1016/j.jim.2012.05.020 22683543

[31] Wang M Saxon A Diaz-Sanchez D. Early IL-4 production driving Th2 differentiation in a human *in vivo* allergic model is mast cell derived. Clin Immunol (1999) 90(1):47–54. doi: 10.1006/clim.1998.4628 9884352

[32] Merz H Kaehler C Hoefig KP Branke B Uckert W Nadrowitz R. Interleukin-9 (IL-9) and NPM-ALK each generate mast cell hyperplasia as single 'hit' and cooperate in producing a mastocytosis-like disease in mice. Oncotarget (2010) 1(2):104–19. doi: 10.18632/oncotarget.115 PMC3157709 21297223

[33] Salomonsson M Dahlin JS Ungerstedt J Hallgren J. Localization-specific expression of CCR1 and CCR5 by mast cell progenitors. Front Immunol (2020) 11:321. doi: 10.3389/fimmu.2020.00321 32174921 PMC7054384

[34] Yu Y Blokhuis B Derks Y Kumari S Garssen J Redegeld F. Human mast cells promote colon cancer growth via bidirectional crosstalk: studies in 2D and 3D coculture models. Oncoimmunology (2018) 7(11):e1504729. doi: 10.1080/2162402X.2018.1504729 30377568 PMC6205014

[35] Kwok E Everingham S Zhang S Greer PA Allingham JS Craig AW. FES kinase promotes mast cell recruitment to mammary tumours via the stem cell factor/KIT receptor signaling axis. Mol Cancer Res (2012) 10(7):881–91. doi: 10.1158/1541-7786.MCR-12-0115 22589410

[36] Huang B Lei Z Zhang GM Li D Song C Li B. SCF-mediated mast cell infiltration and activation exacerbate the inflammation and immunosuppression in tumour microenvironment. Blood (2008) 112(4):1269–79. doi: 10.1182/blood-2008-03-147033 PMC2515142 18524989

[37] Ma Y Hwang RF Logsdon CD Ullrich SE. Dynamic mast cell-stromal cell interactions promote growth of pancreatic cancer. Cancer Res (2013) 73(13):3927–37. doi: 10.1158/0008-5472.CAN-12-4479 PMC3702652 23633481

[38] Lv Y Zhao Y Wang X Chen N Mao F Teng Y. Increased intratumoural mast cells foster immune suppression and gastric cancer progression through TNF-α-PD-L1 pathway. J Immunother Cancer (2019) 7(1):54. doi: 10.1186/s40425-019-0530-3 30808413 PMC6390584

[39] Giannou AD Marazioti A Spella M Kanellakis NI Apostolopoulou H Psallidas I. Mast cells mediate malignant pleural effusion formation. J Clin Invest (2015) 125(6):2317–34. doi: 10.1172/JCI79840 PMC4497757 25915587

[40] Bergot AS Ford N Leggatt GR Wells JW Frazer IH Grimbaldeston MA. HPV16-E7 expression in squamous epithelium creates a local immune suppressive environment via CCL2- and CCL5- mediated recruitment of mast cells. PloS Pathog (2014) 10(10):e1004466. doi: 10.1371/journal.ppat.1004466 25340820 PMC4207828

[41] Zhu XQ Lv JQ Lin Y Xiang M Gao BH Shi YF. Expression of chemokines CCL5 and CCL11 by smooth muscle tumour cells of the uterus and its possible role in the recruitment of mast cells. Gynecol Oncol (2007) 105(3):650–6. doi: 10.1016/j.ygyno.2007.01.046 17368523

[42] Põlajeva J Sjösten AM Lager N Kastemar M Waern I Alafuzoff I. Mast cell accumulation in glioblastoma with a potential role for stem cell factor and chemokine CXCL12. PloS One (2011) 6(9):e25222. doi: 10.1371/journal.pone.0025222 21949886 PMC3176317

[43] Melillo RM Guarino V Avilla E Galdiero MR Liotti F Prevete N. Mast cells have a protumourigenic role in human thyroid cancer. Oncogene (2010) 29(47):6203–15. doi: 10.1038/onc.2010.348 20729915

[44] Segura-Villalobos D Ramírez-Moreno IG Martínez-Aguilar M Ibarra-Sánchez A Muñoz-Bello JO Anaya-Rubio I. Mast cell-tumour interactions: molecular mechanisms of recruitment, intratumoural communication and potential therapeutic targets for tumour growth. Cells (2022) 11(3):349. doi: 10.3390/cells11030349 35159157 PMC8834237

[45] Galli SJ Nakae S Tsai M. Mast cells in the development of adaptive immune responses. Nat Immunol (2005) 6(2):135–42. doi: 10.1038/ni1158 15662442

[46] Cardamone C Parente R Feo GD Triggiani M. Mast cells as effector cells of innate immunity and regulators of adaptive immunity. Immunol Lett (2016) 178:10–4. doi: 10.1016/j.imlet.2016.07.003 27393494

[47] Féger F Varadaradjalou S Gao Z Abraham SN Arock M. The role of mast cells in host defense and their subversion by bacterial pathogens. Trends Immunol (2002) 23(3):151–8. doi: 10.1016/S1471-4906(01)02156-1 11864844

[48] Galli SJ Tsai M Marichal T Tchougounova E Reber LL Pejler G. Approaches for analyzing the roles of mast cells and their proteases *in vivo* . Adv Immunol (2015) 126:45–127. doi: 10.1016/bs.ai.2014.11.002 25727288 PMC4771191

[49] Di Nardo A Vitiello A Gallo RL. Cutting edge: mast cell antimicrobial activity is mediated by expression of cathelicidin antimicrobial peptide. J Immunol (2003) 170(5):2274–8. doi: 10.4049/jimmunol.170.5.2274 12594247

[50] Piliponsky AM Romani L. The contribution of mast cells to bacterial and fungal infection immunity. Immunol Rev (2018) 282(1):188–97. doi: 10.1111/imr.12623 PMC5812373 29431211

[51] De Giovanni M Tam H Valet C Xu Y Looney MR Cyster JG. GPR35 promotes neutrophil recruitment in response to serotonin metabolite 5-HIAA. Cell (2022) 185(5):815–30.e19. doi: 10.1016/j.cell.2022.01.010 35148838 PMC9037118

[52] Triggiani M Granata F Balestrieri B Petraroli A Scalia G Del Vecchio L. Secretory phospholipases A2 activate selective functions in human eosinophils. J Immunol (2003) 170(6):3279–88. doi: 10.4049/jimmunol.170.6.3279 12626587

[53] Triggiani M Gentile M Secondo A Granata F Oriente A Taglialatela M. Histamine induces exocytosis and IL-6 production from human lung macrophages through interaction with H1 receptors. J Immunol (2001) 166(6):4083–91. doi: 10.4049/jimmunol.166.6.4083 11238657

[54] Hepworth MR Maurer M Hartmann S. Regulation of type 2 immunity to helminths by mast cells. Gut Microbes (2012) 3(5):476–81. doi: 10.4161/gmic.21507 PMC3467025 22892692

[55] Dudeck A Köberle M Goldmann O Meyer N Dudeck J Lemmens S. Mast cells as protectors of health. J Allergy Clin Immunol (2019) 144(4s):S4–s18. doi: 10.1016/j.jaci.2018.10.054 30468774

[56] Marichal T Tsai M Galli SJ. Mast cells: potential positive and negative roles in tumour biology. Cancer Immunol Res (2013) 1(5):269–79. doi: 10.1158/2326-6066.CIR-13-0119 24777963

[57] Antsiferova M Martin C Huber M Feyerabend TB Förster A Hartmann K. Mast cells are dispensable for normal and activin-promoted wound healing and skin carcinogenesis. J Immunol (2013) 191(12):6147–55. doi: 10.4049/jimmunol.1301350 24227781

[58] Tuna B Yorukoglu K Unlu M Mungan MU Kirkali Z. Association of mast cells with microvessel density in renal cell carcinomas. Eur Urol (2006) 50(3):530–4. doi: 10.1016/j.eururo.2005.12.040 16426730

[59] Xia Q Wu XJ Zhou Q Jing Z Hou JH Pan ZZ. No relationship between the distribution of mast cells and the survival of stage IIIB colon cancer patients. J Transl Med (2011) 9:88. doi: 10.1186/1479-5876-9-88 21651824 PMC3128057

[60] Molin D Edstrom A Glimelius I Glimelius B Nilsson G Sundstrom C. Mast cell infiltration correlates with poor prognosis in hodgkin's lymphoma. Br J Haematol (2002) 119(1):122–4. doi: 10.1046/j.1365-2141.2002.03768.x 12358914

[61] Englund A Molin D Enblad G Karlen J Glimelius I Ljungman G. The role of tumour-infiltrating eosinophils, mast cells and macrophages in classical and nodular lymphocyte predominant Hodgkin lymphoma in children. Eur J Haematol (2016) 97(5):430–8. doi: 10.1111/ejh.12747 26872637

[62] Andersen MD Kamper P Nielsen PS Bendix K Riber-Hansen R Steiniche T. Tumour-associated mast cells in classical hodgkin's lymphoma: correlation with histological subtype, other tumour-infiltrating inflammatory cell subsets and outcome. Eur J Haematol (2016) 96(3):252–9. doi: 10.1111/ejh.12583 25963595

[63] Molin D Fischer M Xiang Z Larsson U Harvima I Venge P. Mast cells express functional CD30 ligand and are the predominant CD30L-positive cells in hodgkin's disease. Br J Haematol (2001) 114(3):616–23. doi: 10.1046/j.1365-2141.2001.02977.x 11552987

[64] Lv Y Zhao Y Wang X Chen N Mao F Teng Y. Increased intratumoural mast cells foster immune suppression and gastric cancer progression through TNF-alpha-PD-L1 pathway. J Immunother Cancer (2019) 7(1):54. doi: 10.1186/s40425-019-0530-3 30808413 PMC6390584

[65] Ammendola M Sacco R Donato G Zuccala V Russo E Luposella M. Mast cell positivity to tryptase correlates with metastatic lymph nodes in gastrointestinal cancer patients treated surgically. Oncology (2013) 85(2):111–6. doi: 10.1159/000351145 23887206

[66] Lv YP Peng LS Wang QH Chen N Teng YS Wang TT. Degranulation of mast cells induced by gastric cancer-derived adrenomedullin prompts gastric cancer progression. Cell Death Dis (2018) 9(10):1034. doi: 10.1038/s41419-018-1100-1 30305610 PMC6180028

[67] Strouch MJ Cheon EC Salabat MR Krantz SB Gounaris E Melstrom LG. Crosstalk between mast cells and pancreatic cancer cells contributes to pancreatic tumour progression. Clin Cancer Res (2010) 16(8):2257–65. doi: 10.1158/1078-0432.CCR-09-1230 PMC3122919 20371681

[68] Pham L Kennedy L Baiocchi L Meadows V Ekser B Kundu D. Mast cells in liver disease progression: an update on current studies and implications. Hepatology (2022) 75(1):213–8. doi: 10.1002/hep.32121 PMC9276201 34435373

[69] Rao Q Chen Y Yeh CR Ding J Li L Chang C. Recruited mast cells in the tumour microenvironment enhance bladder cancer metastasis via modulation of ERβ/CCL2/CCR2 EMT/MMP9 signals. Oncotarget (2016) 7(7):7842–55. doi: 10.18632/oncotarget.5467 PMC4884958 26556868

[70] Johansson A Rudolfsson S Hammarsten P Halin S Pietras K Jones J. Mast cells are novel independent prognostic markers in prostate cancer and represent a target for therapy. Am J Pathol (2010) 177(2):1031–41. doi: 10.2353/ajpath.2010.100070 PMC2913352 20616342

[71] Fleischmann A Schlomm T Kollermann J Sekulic N Huland H Mirlacher M. Immunological microenvironment in prostate cancer: high mast cell densities are associated with favorable tumour characteristics and good prognosis. Prostate (2009) 69(9):976–81. doi: 10.1002/pros.20948 19274666

[72] Nonomura N Takayama H Nishimura K Oka D Nakai Y Shiba M. Decreased number of mast cells infiltrating into needle biopsy specimens leads to a better prognosis of prostate cancer. Br J Cancer (2007) 97(7):952–6. doi: 10.1038/sj.bjc.6603962 PMC2360404 17848955

[73] Sang J Yi D Tang X Zhang Y Huang T. The associations between mast cell infiltration, clinical features and molecular types of invasive breast cancer. Oncotarget (2016) 7(49):81661–9. doi: 10.18632/oncotarget.13163 PMC5348420 27835573

[74] Amini RM Aaltonen K Nevanlinna H Carvalho R Salonen L Heikkilä P. Mast cells and eosinophils in invasive breast carcinoma. BMC Cancer (2007) 7:165. doi: 10.1186/1471-2407-7-165 17727696 PMC2048965

[75] Dabiri S Huntsman D Makretsov N Cheang M Gilks B Bajdik C. The presence of stromal mast cells identifies a subset of invasive breast cancers with a favorable prognosis. Mod Pathol (2004) 17(6):690–5. doi: 10.1038/modpathol.3800094 15044916

[76] Glajcar A Szpor J Pacek A Tyrak KE Chan F Streb J. The relationship between breast cancer molecular subtypes and mast cell populations in tumour microenvironment. Virchows Arch (2017) 470(5):505–15. doi: 10.1007/s00428-017-2103-5 PMC5406445 28315938

[77] Rajput AB Turbin DA Cheang MC Voduc DK Leung S Gelmon KA. Stromal mast cells in invasive breast cancer are a marker of favourable prognosis: a study of 4,444 cases. Breast Cancer Res Treat (2008) 107(2):249–57. doi: 10.1007/s10549-007-9546-3 PMC2137942 17431762

[78] Marech I Ammendola M Sacco R Capriuolo GS Patruno R Rubini R. Serum tryptase, mast cells positive to tryptase and microvascular density evaluation in early breast cancer patients: possible translational significance. BMC Cancer (2014) 14:534. doi: 10.1186/1471-2407-14-534 25056597 PMC4117953

[79] Xiang M Gu Y Zhao F Lu H Chen S Yin L. Mast cell tryptase promotes breast cancer migration and invasion. Oncol Rep (2010) 23(3):615–9. doi: 10.3892/or_00000676 20126998

[80] Reddy SM Reuben A Barua S Jiang H Zhang S Wang L. Poor response to neoadjuvant chemotherapy correlates with mast cell infiltration in inflammatory breast cancer. Cancer Immunol Res (2019) 7(6):1025–35. doi: 10.1158/2326-6066.CIR-18-0619 PMC7053657 31043414

[81] Polyak K. Heterogeneity in breast cancer. J Clin Invest (2011) 121(10):3786–8. doi: 10.1172/JCI60534 PMC3195489 21965334

[82] Imada A Shijubo N Kojima H Abe S. Mast cells correlate with angiogenesis and poor outcome in stage I lung adenocarcinoma. Eur Respir J (2000) 15(6):1087–93. doi: 10.1034/j.1399-3003.2000.01517.x 10885428

[83] Carlini MJ Dalurzo MC Lastiri JM Smith DE Vasallo BC Puricelli LI. Mast cell phenotypes and microvessels in non-small cell lung cancer and its prognostic significance. Hum Pathol (2010) 41(5):697–705. doi: 10.1016/j.humpath.2009.04.029 20040391

[84] Varricchi G Galdiero MR Marone G Granata F Borriello F Marone G. Controversial role of mast cells in skin cancers. Exp Dermatol (2017) 26(1):11–7. doi: 10.1111/exd.13107 27305467

[85] Siiskonen H Poukka M Bykachev A Tyynela-Korhonen K Sironen R Pasonen-Seppanen S. Low numbers of tryptase+ and chymase+ mast cells associated with reduced survival and advanced tumour stage in melanoma. Melanoma Res (2015) 25(6):479–85. doi: 10.1097/CMR.0000000000000192 26317168

[86] Hölzel M Landsberg J Glodde N Bald T Rogava M Riesenberg S. A preclinical model of malignant peripheral nerve sheath tumour-like melanoma is characterized by infiltrating mast cells. Cancer Res (2016) 76(2):251–63. doi: 10.1158/0008-5472.CAN-15-1090 26511633

[87] Somasundaram R Connelly T Choi R Choi H Samarkina A Li L. Tumour-infiltrating mast cells are associated with resistance to anti-PD-1 therapy. Nat Commun (2021) 12(1):346. doi: 10.1038/s41467-020-20600-7 33436641 PMC7804257

[88] Martinez-Corral I Olmeda D Dieguez-Hurtado R Tammela T Alitalo K Ortega S. *In vivo* imaging of lymphatic vessels in development, wound healing, inflammation, and tumour metastasis. Proc Natl Acad Sci U S A (2012) 109(16):6223–8. doi: 10.1073/pnas.1115542109 PMC3341065 22474390

[89] Varricchi G Granata F Loffredo S Genovese A Marone G. Angiogenesis and lymphangiogenesis in inflammatory skin disorders. J Am Acad Dermatol (2015) 73(1):144–53. doi: 10.1016/j.jaad.2015.03.041 25922287

[90] Loffredo S Staiano RI Granata F Genovese A Marone G. Immune cells as a source and target of angiogenic and lymphangiogenic factors. Chem Immunol Allergy (2014) 99:15–36. doi: 10.1159/000353316 24217601

[91] Folkman J. Tumour angiogenesis: therapeutic implications. N Engl J Med (1971) 285(21):1182–6. doi: 10.1056/NEJM197111182852108 4938153

[92] Folkman J Shing Y. Angiogenesis. J Biol Chem (1992) 267(16):10931–4. doi: 10.1016/S0021-9258(19)49853-0 1375931

[93] Dieterich LC Tacconi C Ducoli L Detmar M. Lymphatic vessels in cancer. Physiol Rev (2022) 102(4):1837–79. doi: 10.1152/physrev.00039.2021 35771983

[94] Detoraki A Staiano RI Granata F Giannattasio G Prevete N de Paulis A. Vascular endothelial growth factors synthesized by human lung mast cells exert angiogenic effects. J Allergy Clin Immunol (2009) 123(5):1142–9, 9.e1-5. doi: 10.1016/j.jaci.2009.01.044 19275959

[95] Sismanopoulos N Delivanis DA Alysandratos KD Angelidou A Vasiadi M Therianou A. IL-9 induces VEGF secretion from human mast cells and IL-9/IL-9 receptor genes are overexpressed in atopic dermatitis. PloS One (2012) 7(3):e33271. doi: 10.1371/journal.pone.0033271 22413008 PMC3297631

[96] Boesiger J Tsai M Maurer M Yamaguchi M Brown LF Claffey KP. Mast cells can secrete vascular permeability factor/ vascular endothelial cell growth factor and exhibit enhanced release after immunoglobulin e-dependent upregulation of fc epsilon receptor I expression. J Exp Med (1998) 188(6):1135–45. doi: 10.1084/jem.188.6.1135 PMC2212544 9743532

[97] Grützkau A Krüger-Krasagakes S Baumeister H Schwarz C Kögel H Welker P. Synthesis, storage, and release of vascular endothelial growth factor/vascular permeability factor (VEGF/VPF) by human mast cells: implications for the biological significance of VEGF206. Mol Biol Cell (1998) 9(4):875–84. doi: 10.1091/mbc.9.4.875 PMC25314 9529385

[98] Simons M Gordon E Claesson-Welsh L. Mechanisms and regulation of endothelial VEGF receptor signalling. Nat Rev Mol Cell Biol (2016) 17(10):611–25. doi: 10.1038/nrm.2016.87 27461391

[99] Detoraki A Granata F Staibano S Rossi FW Marone G Genovese A. Angiogenesis and lymphangiogenesis in bronchial asthma. Allergy (2010) 65(8):946–58. doi: 10.1111/j.1398-9995.2010.02372.x 20415716

[100] Karaman S Leppanen VM Alitalo K. Vascular endothelial growth factor signaling in development and disease. Development (2018) 145(14):dev151019. doi: 10.1242/dev.151019 30030240

[101] Leppanen VM Tvorogov D Kisko K Prota AE Jeltsch M Anisimov A. Structural and mechanistic insights into VEGF receptor 3 ligand binding and activation. Proc Natl Acad Sci U S A (2013) 110(32):12960–5. doi: 10.1073/pnas.1301415110 PMC3740881 23878260

[102] Huang H Bhat A Woodnutt G Lappe R. Targeting the ANGPT-TIE2 pathway in malignancy. Nat Rev Cancer (2010) 10(8):575–85. doi: 10.1038/nrc2894 20651738

[103] Thomas M Augustin HG. The role of the angiopoietins in vascular morphogenesis. Angiogenesis (2009) 12(2):125–37. doi: 10.1007/s10456-009-9147-3 19449109

[104] Eklund L Kangas J Saharinen P. Angiopoietin-tie signalling in the cardiovascular and lymphatic systems. Clin Sci (Lond) (2017) 131(1):87–103. doi: 10.1042/CS20160129 27941161 PMC5146956

[105] Yuan HT Khankin EV Karumanchi SA Parikh SM. Angiopoietin 2 is a partial agonist/antagonist of Tie2 signaling in the endothelium. Mol Cell Biol (2009) 29(8):2011–22. doi: 10.1128/MCB.01472-08 PMC2663314 19223473

[106] Longo V Catino A Montrone M Galetta D Ribatti D. Controversial role of mast cells in NSCLC tumour progression and angiogenesis. Thorac Cancer (2022) 13(21):2929–34. doi: 10.1111/1759-7714.14654 PMC9626321 36196487

[107] Prevete N Staiano RI Granata F Detoraki A Necchi V Ricci V. Expression and function of angiopoietins and their tie receptors in human basophils and mast cells. J Biol Regul Homeost Agents (2013) 27(3):827–39. 24152847

[108] Ribatti D Ranieri G Basile A Azzariti A Paradiso A Vacca A. Tumour endothelial markers as a target in cancer. Expert Opin Ther Targets (2012) 16(12):1215–25. doi: 10.1517/14728222.2012.725047 22978444

[109] Hassan S Kinoshita Y Kawanami C Kishi K Matsushima Y Ohashi A. Expression of protooncogene c-kit and its ligand stem cell factor (SCF) in gastric carcinoma cell lines. Dig Dis Sci (1998) 43(1):8–14. doi: 10.1023/A:1018851415704 9508539

[110] Ribatti D Ranieri G Nico B Benagiano V Crivellato E. Tryptase and chymase are angiogenic *in vivo* in the chorioallantoic membrane assay. Int J Dev Biol (2011) 55(1):99–102. doi: 10.1387/ijdb.103138dr 21425085

[111] Aponte-López A Muñoz-Cruz S. Mast cells in the tumour microenvironment. Adv Exp Med Biol (2020) 1273:159–73. doi: 10.1007/978-3-030-49270-0_9 33119881

[112] Macfarlane SR Seatter MJ Kanke T Hunter GD Plevin R. Proteinase-activated receptors. Pharmacol Rev (2001) 53(2):245–82. 11356985

[113] Ducroc R Bontemps C Marazova K Devaud H Darmoul D Laburthe M. Trypsin is produced by and activates protease-activated receptor-2 in human cancer colon cells: evidence for new autocrine loop. Life Sci (2002) 70(12):1359–67. doi: 10.1016/S0024-3205(01)01519-3 11883712

[114] Liu Y Mueller BM. Protease-activated receptor-2 regulates vascular endothelial growth factor expression in MDA-MB-231 cells via MAPK pathways. Biochem Biophys Res Commun (2006) 344(4):1263–70. doi: 10.1016/j.bbrc.2006.04.005 16650817

[115] Kim GY Lee JW Ryu HC Wei JD Seong CM Kim JH. Proinflammatory cytokine IL-1beta stimulates IL-8 synthesis in mast cells via a leukotriene B4 receptor 2-linked pathway, contributing to angiogenesis. J Immunol (2010) 184(7):3946–54. doi: 10.4049/jimmunol.0901735 20194723

[116] Feoktistov I Ryzhov S Goldstein AE Biaggioni I. Mast cell-mediated stimulation of angiogenesis: cooperative interaction between A2B and A3 adenosine receptors. Circ Res (2003) 92(5):485–92. doi: 10.1161/01.RES.0000061572.10929.2D 12600879

[117] Mehdawi L Osman J Topi G Sjölander A. High tumour mast cell density is associated with longer survival of colon cancer patients. Acta Oncol (2016) 55(12):1434–42. doi: 10.1080/0284186X.2016.1198493 27355473

[118] Elezoğlu B Tolunay S. The relationship between the stromal mast cell number, microvessel density, c-erbB-2 staining and survival and prognostic factors in colorectal carcinoma. Turk Patoloji Derg (2012) 28(2):110–8. doi: 10.5146/tjpath.2012.01109 22627628

[119] Xia Q Ding Y Wu XJ Peng RQ Zhou Q Zeng J. Mast cells in adjacent normal colon mucosa rather than those in invasive margin are related to progression of colon cancer. Chin J Cancer Res (2011) 23(4):276–82. doi: 10.1007/s11670-011-0276-z PMC3551301 23358806

[120] Zhao P Zhou P Tang T Si R Ji Y Hu X. Levels of circulating mast cell progenitors and tumour−infiltrating mast cells in patients with colorectal cancer. Oncol Rep (2022) 47(5):89. doi: 10.3892/or.2022.8300 35293596 PMC8931805

[121] Suzuki S Ichikawa Y Nakagawa K Kumamoto T Mori R Matsuyama R. High infiltration of mast cells positive to tryptase predicts worse outcome following resection of colorectal liver metastases. BMC Cancer (2015) 15:840. doi: 10.1186/s12885-015-1863-z 26530140 PMC4632336

[122] Malfettone A Silvestris N Saponaro C Ranieri G Russo A Caruso S. High density of tryptase-positive mast cells in human colorectal cancer: a poor prognostic factor related to protease-activated receptor 2 expression. J Cell Mol Med (2013) 17(8):1025–37. doi: 10.1111/jcmm.12073 PMC3780541 23991686

[123] Mao Y Feng Q Zheng P Yang L Zhu D Chang W. Low tumour infiltrating mast cell density confers prognostic benefit and reflects immunoactivation in colorectal cancer. Int J Cancer (2018) 143(9):2271–80. doi: 10.1002/ijc.31613 29873076

[124] Gulubova M Vlaykova T. Prognostic significance of mast cell number and microvascular density for the survival of patients with primary colorectal cancer. J Gastroenterol Hepatol (2009) 24(7):1265–75. doi: 10.1111/j.1440-1746.2007.05009.x 17645466

[125] Fisher ER Paik SM Rockette H Jones J Caplan R Fisher B. Prognostic significance of eosinophils and mast cells in rectal cancer: findings from the national surgical adjuvant breast and bowel project (protocol r-01). Hum Pathol (1989) 20(2):159–63. doi: 10.1016/0046-8177(89)90180-9 2562788

[126] Molderings GJ Zienkiewicz T Homann J Menzen M Afrin LB. Risk of solid cancer in patients with mast cell activation syndrome: results from Germany and USA. F1000Res (2017) 6:1889. doi: 10.12688/f1000research.12730.1 29225779 PMC5710302

[127] Dwyer DF Barrett NA Austen KF. Expression profiling of constitutive mast cells reveals a unique identity within the immune system. Nat Immunol (2016) 17(7):878–87. doi: 10.1038/ni.3445 PMC5045264 27135604

[128] Flores de Los Rios PA Soto Dominguez A Arellano-Perez Vertti RD Castelan Maldonado EE Velazquez Gauna SE Moran Martinez J. Differential expression of mast cell granules in samples of metastatic and non-metastatic colorectal cancer in patients. Acta Histochem (2020) 122(7):151618. doi: 10.1016/j.acthis.2020.151618 33066840

[129] Leveque E Rouch A Syrykh C Mazieres J Brouchet L Valitutti S. Phenotypic and histological distribution analysis identify mast cell heterogeneity in non-small cell lung cancer. Cancers (Basel) (2022) 14(6):1394. doi: 10.3390/cancers14061394 35326546 PMC8946292

[130] Sakita JY Elias-Oliveira J Carlos D de Souza Santos E Almeida LY Malta TM. Mast cell-T cell axis alters development of colitis-dependent and colitis-independent colorectal tumours: potential for therapeutically targeting via mast cell inhibition. J Immunother Cancer (2022) 10(10):e004653. doi: 10.1136/jitc-2022-004653 36220303 PMC9557261

[131] Danelli L Frossi B Gri G Mion F Guarnotta C Bongiovanni L. Mast cells boost myeloid-derived suppressor cell activity and contribute to the development of tumour-favoring microenvironment. Cancer Immunol Res (2015) 3(1):85–95. doi: 10.1158/2326-6066.CIR-14-0102 25351848

[132] Maywald RL Doerner SK Pastorelli L De Salvo C Benton SM Dawson EP. IL-33 activates tumour stroma to promote intestinal polyposis. Proc Natl Acad Sci U.S.A. (2015) 112(19):E2487–96. doi: 10.1073/pnas.1422445112 PMC4434739 25918379

[133] Yu Y Blokhuis BRJ Diks MAP Keshavarzian A Garssen J Redegeld FA. Functional inhibitory siglec-6 is upregulated in human colorectal cancer-associated mast cells. Front Immunol (2018) 9:2138. doi: 10.3389/fimmu.2018.02138 30294327 PMC6159741

[134] Ali HR Provenzano E Dawson SJ Blows FM Liu B Shah M. Association between CD8+ T-cell infiltration and breast cancer survival in 12,439 patients. Ann Oncol (2014) 25(8):1536–43. doi: 10.1093/annonc/mdu191 24915873

[135] Feng Q Chang W Mao Y He G Zheng P Tang W. Tumour-associated macrophages as prognostic and predictive biomarkers for postoperative adjuvant chemotherapy in patients with stage II colon cancer. Clin Cancer Res (2019) 25(13):3896–907. doi: 10.1158/1078-0432.CCR-18-2076 30988081

[136] Wang M Zhao J Zhang L Wei F Lian Y Wu Y. Role of tumour microenvironment in tumourigenesis. J Cancer (2017) 8(5):761–73. doi: 10.7150/jca.17648 PMC5381164 28382138

[137] Zhang X Quan F Xu J Xiao Y Li X Li Y. Combination of multiple tumour-infiltrating immune cells predicts clinical outcome in colon cancer. Clin Immunol (2020) 215:108412. doi: 10.1016/j.clim.2020.108412 32278085

[138] Yoshii M Jikuhara A Mori S Iwagaki H Takahashi HK Nishibori M. Mast cell tryptase stimulates DLD-1 carcinoma through prostaglandin- and MAP kinase-dependent manners. J Pharmacol Sci (2005) 98(4):450–8. doi: 10.1254/jphs.FPJ05002X 16093613

[139] Ranieri G Gadaleta CD Patruno R Zizzo N Daidone MG Hansson MG. A model of study for human cancer: spontaneous occurring tumours in dogs. biological features and translation for new anticancer therapies. Crit Rev Oncol Hematol (2013) 88(1):187–97. doi: 10.1016/j.critrevonc.2013.03.005 23561333

[140] Ranieri G Pantaleo M Piccinno M Roncetti M Mutinati M Marech I. Tyrosine kinase inhibitors (TKIs) in human and pet tumours with special reference to breast cancer: a comparative review. Crit Rev Oncol Hematol (2013) 88(2):293–308. doi: 10.1016/j.critrevonc.2013.05.009 23768779

[141] Kajiguchi T Lee S Lee MJ Trepel JB Neckers L. KIT regulates tyrosine phosphorylation and nuclear localization of beta-catenin in mast cell leukemia. Leuk Res (2008) 32(5):761–70. doi: 10.1016/j.leukres.2007.08.023 PMC2682210 17949810

[142] Voloshanenko O Erdmann G Dubash TD Augustin I Metzig M Moffa G. Wnt secretion is required to maintain high levels of wnt activity in colon cancer cells. Nat Commun (2013) 4:2610. doi: 10.1038/ncomms3610 24162018 PMC3826636

[143] Saadalla A Lima MM Tsai F Osman A Singh MP Linden DR. Cell intrinsic deregulated ss-catenin signaling promotes expansion of bone marrow derived connective tissue type mast cells, systemic inflammation, and colon cancer. Front Immunol (2019) 10:2777. doi: 10.3389/fimmu.2019.02777 31849960 PMC6902090

[144] Jin B Ding K Pan J. Ponatinib induces apoptosis in imatinib-resistant human mast cells by dephosphorylating mutant D816V KIT and silencing β-catenin signaling. Mol Cancer Ther (2014) 13(5):1217–30. doi: 10.1158/1535-7163.MCT-13-0397 24552773

[145] Xu L Yi HG Wu Z Han W Chen K Zang M. Activation of mucosal mast cells promotes inflammation-related colon cancer development through recruiting and modulating inflammatory CD11b(+)Gr1(+) cells. Cancer Lett (2015) 364(2):173–80. doi: 10.1016/j.canlet.2015.05.014 25986744

[146] Huang B Lei Z Zhao J Gong W Liu J Chen Z. CCL2/CCR2 pathway mediates recruitment of myeloid suppressor cells to cancers. Cancer Lett (2007) 252(1):86–92. doi: 10.1016/j.canlet.2006.12.012 17257744

[147] Wang S Li L Shi R Liu X Zhang J Zou Z. Mast cell targeted chimeric toxin can be developed as an adjunctive therapy in colon cancer treatment. Toxins (Basel) (2016) 8(3):71. doi: 10.3390/toxins8030071 26978404 PMC4810216

[148] Lin MT Lin BR Chang CC Chu CY Su HJ Chen ST. IL-6 induces AGS gastric cancer cell invasion via activation of the c-Src/RhoA/ROCK signaling pathway. Int J Cancer (2007) 120(12):2600–8. doi: 10.1002/ijc.22599 17304514

[149] Kim EK Choi EJ. Pathological roles of MAPK signaling pathways in human diseases. Biochim Biophys Acta (2010) 1802(4):396–405. doi: 10.1016/j.bbadis.2009.12.009 20079433

[150] Taylor CA Zheng Q Liu Z Thompson JE. Role of p38 and JNK MAPK signaling pathways and tumour suppressor p53 on induction of apoptosis in response to ad-eIF5A1 in A549 lung cancer cells. Mol Cancer (2013) 12:35. doi: 10.1186/1476-4598-12-35 23638878 PMC3660295

[151] Liang X Yin G Ma Y Xu K Liu J Li J. The critical role of mast cell-derived hypoxia-inducible factor-1alpha in regulating mast cell function. J Pharm Pharmacol (2016) 68(11):1409–16. doi: 10.1111/jphp.12622 27671226

[152] Yu Y Blokhuis BR Garssen J Redegeld FA. A transcriptomic insight into the impact of colon cancer cells on mast cells. Int J Mol Sci (2019) 20(7):1689. doi: 10.3390/ijms20071689 30987352 PMC6480031

[153] Lopetuso LR Scaldaferri F Pizarro TT. Emerging role of the interleukin (IL)-33/ST2 axis in gut mucosal wound healing and fibrosis. Fibrogenesis Tissue Repair (2012) 5(1):18. doi: 10.1186/1755-1536-5-18 23062310 PMC3514189

[154] Wood IS Wang B Trayhurn P. IL-33, a recently identified interleukin-1 gene family member, is expressed in human adipocytes. Biochem Biophys Res Commun (2009) 384(1):105–9. doi: 10.1016/j.bbrc.2009.04.081 19393621

[155] Moussion C Ortega N Girard JP. The IL-1-like cytokine IL-33 is constitutively expressed in the nucleus of endothelial cells and epithelial cells *in vivo*: a novel 'alarmin'? PloS One (2008) 3(10):e3331. doi: 10.1371/journal.pone.0003331 18836528 PMC2556082

[156] Macauley MS Crocker PR Paulson JC. Siglec-mediated regulation of immune cell function in disease. Nat Rev Immunol (2014) 14(10):653–66. doi: 10.1038/nri3737 PMC4191907 25234143

[157] Yokoi H Myers A Matsumoto K Crocker PR Saito H Bochner BS. Alteration and acquisition of siglecs during *in vitro* maturation of CD34+ progenitors into human mast cells. Allergy (2006) 61(6):769–76. doi: 10.1111/j.1398-9995.2006.01133.x 16677248

[158] Mizrahi S Gibbs BF Karra L Ben-Zimra M Levi-Schaffer F. Siglec-7 is an inhibitory receptor on human mast cells and basophils. J Allergy Clin Immunol (2014) 134(1):230–3. doi: 10.1016/j.jaci.2014.03.031 24810846

[159] Yazawa T Shibata M Gonda K Machida T Suzuki S Kenjo A. Increased IL-17 production correlates with immunosuppression involving myeloid-derived suppressor cells and nutritional impairment in patients with various gastrointestinal cancers. Mol Clin Oncol (2013) 1(4):675–9. doi: 10.3892/mco.2013.134 PMC3916208 24649227

[160] Taams LS Steel KJA Srenathan U Burns LA Kirkham BW. IL-17 in the immunopathogenesis of spondyloarthritis. Nat Rev Rheumatol (2018) 14(8):453–66. doi: 10.1038/s41584-018-0044-2 30006601

[161] Kim HY Lee HJ Chang YJ Pichavant M Shore SA Fitzgerald KA. Interleukin-17-producing innate lymphoid cells and the NLRP3 inflammasome facilitate obesity-associated airway hyperreactivity. Nat Med (2014) 20(1):54–61. doi: 10.1038/nm.3423 24336249 PMC3912313

[162] Kim JS Jordan MS. Diversity of IL-17-producing T lymphocytes. Cell Mol Life Sci (2013) 70(13):2271–90. doi: 10.1007/s00018-012-1163-6 PMC3568230 23052209

[163] Chen X Churchill MJ Nagar KK Tailor YH Chu T Rush BS. IL-17 producing mast cells promote the expansion of myeloid-derived suppressor cells in a mouse allergy model of colorectal cancer. Oncotarget (2015) 6(32):32966–79. doi: 10.18632/oncotarget.5435 PMC4741743 26429861

[164] Iwanaga K Nakamura T Maeda S Aritake K Hori M Urade Y. Mast cell-derived prostaglandin D2 inhibits colitis and colitis-associated colon cancer in mice. Cancer Res (2014) 74(11):3011–9. doi: 10.1158/0008-5472.CAN-13-2792 24879565

[165] Mussap M Plebani M. Biochemistry and clinical role of human cystatin c. Crit Rev Clin Lab Sci (2004) 41(5-6):467–550. doi: 10.1080/10408360490504934 15603510

[166] Xu Y Ding Y Li X Wu X. Cystatin c is a disease-associated protein subject to multiple regulation. Immunol Cell Biol (2015) 93(5):442–51. doi: 10.1038/icb.2014.121 PMC7165929 25643616

